# An aquaculture simulator for rainbow trout (*Oncorhynchus mykiss*) based on a fish schooling behavioral model and a dynamic energy budget

**DOI:** 10.1038/s41598-026-39028-y

**Published:** 2026-02-07

**Authors:** Yuki Takahashi, Taishu Yoshida, Yuto Yamazaki, Eisuke Takahashi, Etsurou Yamaha, Kazuyoshi Komeyama

**Affiliations:** 1 Faculty of Fisheries Sciences, Hokkaido University, 3-1-1, Minato-cho, Hakodate, 041-8611 Hokkaido Japan; 2 Graduate School of Fisheries Sciences, Hokkaido University, 3-1-1, Minato- cho, Hakodate, 041-8611 Hokkaido Japan; 3 Field Science Center for Northern Biosphere, Nanae Fresh-Water Station, Hokkaido University, 2-9-1, Sakuracho, Nanae town Kameda-gun, Hokkaido, 041-1105 Japan

**Keywords:** Aquaculture decision making, Feeding strategy, Fish behavior model, Growth model, Numerical simulation, Rainbow trout, Ecology, Ecology, Zoology

## Abstract

Aquaculture is increasingly important for meeting the rising global demand for seafood. To improve the sustainability of the aquaculture industry, there have been increasing efforts to develop smart aquaculture technologies, including simulation methods, to optimize growth and feeding strategies. This study developed a simulation model, which incorporates a fish behavior model based on the Boids model and a dynamic energy budget, for the purpose of improving the efficiency of rainbow trout (*Oncorhynchus mykiss*) aquaculture. The proposed simulation method predicts the growth trajectories of individual fish and evaluates the effects of different feeding levels on fish growth and feed efficiency. The simulation results were compared with those of a live rearing experiment to evaluate its accuracy. Rainbow trout growth trajectories were accurately predicted. However, longer-term simulations showed increasing divergence between the simulated and experimental data. The proposed simulation method allows the optimization of growth and feeding efficiency under various feeding strategies. Further refinements of the simulation model, including considering density effects and parameter adjustments, may lead to more accurate long-term predictions. The simulation-based approach developed in this study will contribute to a better understanding of rainbow trout growth, with potential applications for other aquaculture species and contexts.

**Supplementary Information:**

The online version contains supplementary material available at 10.1038/s41598-026-39028-y.

## Introduction

The global demand for fish has grown dramatically in recent years. Concurrently, aquaculture production has been increasing steadily, exceeding fishing vessel production^1^. In recent years, there has been a decline in natural fishery resources, making aquaculture increasingly important for fisheries production. In Japan, there has been growing interest in innovative aquaculture production technologies, particularly in the development of “smart aquaculture” systems, which couple information and communications technology with Internet of Things applications.

In Japan, most aquaculture production is conducted at sea, and accounts for approximately 97% of the total aquaculture output^2^. Marine aquaculture can only be established in relatively calm coastal areas, and most of the locations suitable for aquaculture in Japan are already in use. Marine aquaculture faces other challenges, such as concerns about eutrophication in the surrounding waters due to the direct discharge of residual feed and waste. Such factors make it difficult to significantly increase marine aquaculture production. Therefore, the focus has shifted from marine aquaculture to land-based aquaculture, in which fish are farmed in tanks on land. While the land-based aquaculture sites need to acquire land area, they are not restricted by the availability of suitable locations at sea and pose less risk of eutrophication compared to marine aquaculture. Furthermore, land-based aquaculture has the advantage of consistently stable water quality and is not affected by natural environmental variability. However, land-based aquaculture is less profitable due to higher costs and income instability.

The greatest cost associated with any type of aquaculture is related to feed. Feed costs can account for around 60% of the total aquaculture costs^3^. Land-based aquaculture has additional expenses such as electricity and facility construction^4,5^, which make it inherently less profitable than marine aquaculture. Because aquaculture farming income is calculated based on the reared fish mass, it is dependent on the growth rate of the farmed fish. Various studies have experimentally evaluated the impact of external parameters on fish growth^6^. For example, Handeland et al.^7^ performed a rearing experiment targeting Atlantic salmon (*Salmo salar*) at different water temperatures to determine the optimal water temperature for maximal feed conversion efficiency. Previous studies have also evaluated the optimal feeding efficiency for the rainbow trout, *Oncorhynchus mykiss*. For example, Azevedo et al.^8^ investigated the effects of feeding level and water temperature on fish growth and feeding efficiency. Bureau et al.^9^ focused on the feeding level when conducting rearing experiments at various feeding levels (25–100%). The cited authors evaluated differences in growth by various nutrient compositions. Fish growth rates are influenced by the amount of feed provided, which in turn affects the total feed cost. Therefore, to maximize aquaculture profitability, it is necessary to determine the most cost-effective feeding amount. Traditionally, optimal feed conversion efficiency has been determined only through live rearing experiments (e.g., Azevedo et al.^8^. However, these experiments are time-consuming and costly. Therefore, there has been growing interest in simulation studies to determine optimal aquaculture operations parameters, such as feeding efficiency, without performing actual fish rearing experiments.

Dynamic energy budget (DEB) models^10^ have been widely applied to estimate growth rates in many animals, including fish. For example, Alver et al.^11^ applied a DEB to larval-stage Atlantic cod *Gadus morhua*. A simulation model incorporating a fish behavioral model and a DEB, developed to estimate the growth of Atlantic salmon, was applied in a series of subsequent studies including simulations of mean body mass during fish growth^12–15^. Although these studies yielded important insights, most evaluations were at the group level (e.g., mean body mass). A similar, but more recent study by Takahashi and Komeyama^16^ developed a swimming behavior simulation model, based on the Boids model^17^, to predict growth changes in individual fish.

In this study, we developed an aquaculture simulation method, which couples an established fish behavior model^16^ and a DEB, to evaluate growth differences in rainbow trout based on the feeding patterns. Next, we performed a live rainbow trout rearing experiment. We measured body length and mass. We verified the accuracy of the simulation by comparing its results with those of the rearing experiment and used the findings to explore the practicality of the developed aquaculture simulation model.

## Methods

### Numerical simulation model

We used rainbow trout in this study. The proposed simulation approach was divided into fish behavior and growth simulation components. In the fish behavior simulation component, fish behavior including typical swimming and feeding actions were simulated based on the Boids model^17^. This is an individual-based interaction model that was employed in a previous study to simulate the movement of rainbow trout^16^. In the fish growth component, the feed intake of each individual is simulated, and this information is passed to the growth model. The details are explained below.

#### Fish behavior simulation

Fish schooling behavior is determined by interference from other individuals. Several models have been developed to evaluate fish school movements from individual behavior, such as the Boids model^17^, the Sannomiya model^18,19^, and stochastic models^20^. In this study, we used a previously developed feeding simulation model of behavior based on the Boids model^16^:

$$\:{W}_{n}^{i}{\boldsymbol{a}}_{t+\varDelta\:t}={\boldsymbol{F}}_{t}$$

1 $$\:{\boldsymbol{v}}_{t+\varDelta\:t}={\boldsymbol{v}}_{t}+{\boldsymbol{a}}_{t+\varDelta\:t}\bullet\:\varDelta\:t$$

$$\:{\boldsymbol{x}}_{t+\varDelta\:t}={\boldsymbol{x}}_{t}+{\boldsymbol{v}}_{t+\varDelta\:t}\bullet\:\varDelta\:t$$

where $\:{W}_{n}^{i}$ is the fish body mass, ***a***_*t*_, ***v***_*t*_, ***x***_*t*_, and ***F***_*t*_ are the acceleration, velocity, position, and swimming force vectors at time *t*, respectively, and Δ*t* is the time step of the simulation (0.025 s).

**Fig. 1:**
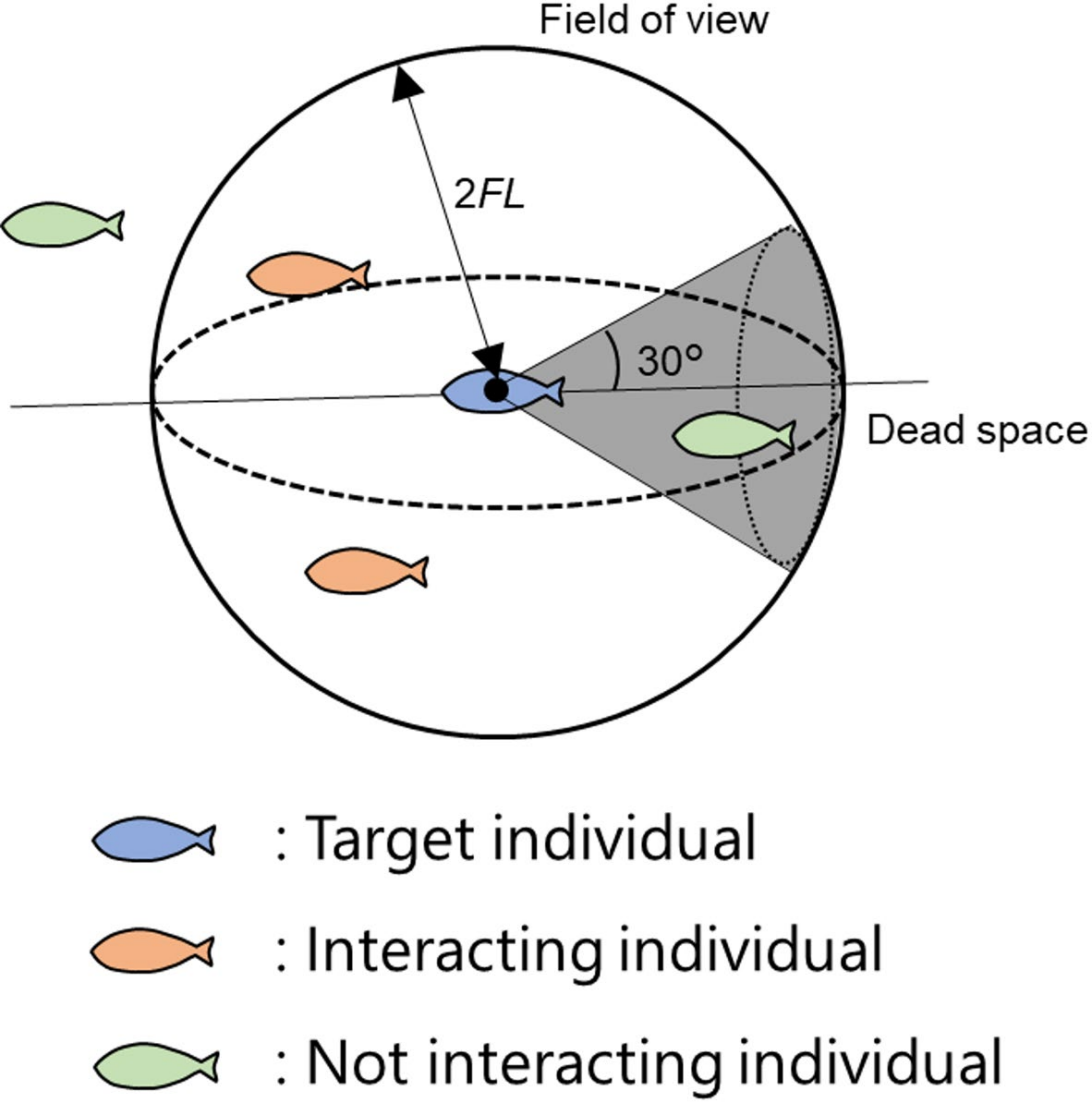
Schematic of an individual fish, its field of view, and its interactions with other individuals^16^.

In Eq. (1), ***F***_*t*_ is determined by the interference of neighboring individuals. Figure 1 presents a schematic of the motion of an individual modeled fish. The ***F***_*t*_ of the fish is determined by other individuals within the field of view, which is assumed to be a simple sphere with a radius twice the fork length (2 FL), where the space behind the fish within an angle of 30° is designated as “dead space”.

**Fig. 2:**
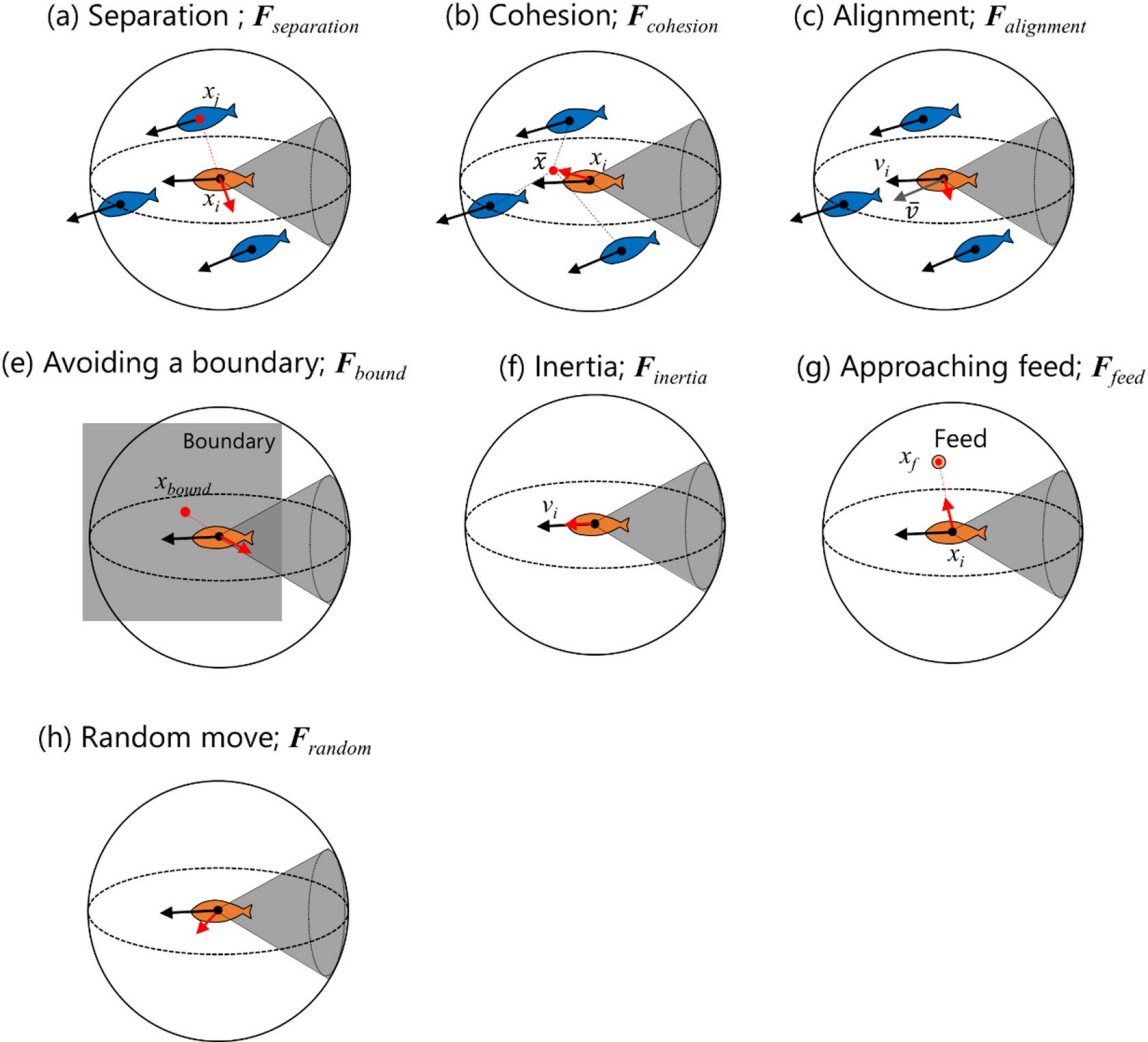
Overview of the force vectors of the proposed fish behavior model^16^.

An overview of the swimming force of an individual fish is provided in Fig. 2. In this study, fish schooling behavior was assumed to be governed by seven rules: separation (***F***_*separation*_), cohesion (***F***_*cohesion*_), alignment (***F***_*alignment*_), boundary avoidance (***F***_*bound*_), inertia (***F***_*inertia*_), approaching feed (***F***_*feed*_), and random movement (***F***_*random*_). Thus, the swimming force is calculated:

2 $$\:{\boldsymbol{F}}_{t}=\sum\:_{n=1}^{7}{\boldsymbol{F}}_{n}$$

where ***F***_*n*_ is the *n*^th^ force vector, expressed^16^:

3 $$\:{\boldsymbol{F}}_{separation}={w}_{separation}\frac{{\boldsymbol{x}}_{i}-{\boldsymbol{x}}_{j}}{\left|{\boldsymbol{x}}_{i}-{\boldsymbol{x}}_{j}\right|}$$

4 $$\:{\boldsymbol{F}}_{cohesion}={w}_{cohesion}\frac{{\stackrel{-}{\boldsymbol{x}}-\boldsymbol{x}}_{i}}{\left|{\stackrel{-}{\boldsymbol{x}}-\boldsymbol{x}}_{i}\right|}$$

5 $$\:{\boldsymbol{F}}_{alignment}={w}_{alignment}\frac{{\stackrel{-}{\boldsymbol{v}}-\boldsymbol{v}}_{i}}{\left|{\stackrel{-}{\boldsymbol{v}}-\boldsymbol{v}}_{i}\right|}$$

6 $$\:{\boldsymbol{F}}_{bound}={w}_{bound}\frac{{\boldsymbol{x}}_{i}-{\boldsymbol{x}}_{bound}}{\left|{\boldsymbol{x}}_{i}-{\boldsymbol{x}}_{bound}\right|}$$

7 $$\:{\boldsymbol{F}}_{inertia}={w}_{inertia}\frac{{\boldsymbol{v}}_{i}}{\left|{\boldsymbol{v}}_{i}\right|}$$

8 $$\:{\boldsymbol{F}}_{feed}={w}_{feed}\frac{{\boldsymbol{x}}_{f}-{\boldsymbol{x}}_{i}}{\left|{\boldsymbol{x}}_{f}-{\boldsymbol{x}}_{i}\right|}$$

9 $$\:{\boldsymbol{F}}_{random}=\frac{{w}_{random}}{\sqrt{{{x}_{1}}^{2}+{{x}_{2}}^{2}+{{x}_{3}}^{2}}}{\left({x}_{1},\:{x}_{2},{x}_{3}\right)}^{T}$$

where ***x***_*i*_ is the position vector of the individual, ***x***_*j*_ is the position vector of the nearest other individual, ***v***_*i*_ is the velocity vector of the individual, ***x***_*bound*_ is the position vector of the nearest boundary point in the field of view, ***x***_*f*_ is the position vector of the feed, ***x***_*1*_, ***x***_*2*_, and ***x***_*3*_ are random numbers uniformly distributed over the range [–1, 1], *w*_*n*_ is the weight coefficient of ***F***_*n*_, and $\:\stackrel{-}{\boldsymbol{x}}$.

is the center of gravity position vector of other individuals in the field of view, which is calculated:

10 $$\:\stackrel{-}{\boldsymbol{x}}=\frac{\sum\:_{k=1}^{m}{\boldsymbol{x}}_{k}}{m}$$

where ***x***_*k*_ is the position vector of the *k*^th^ individual in the field of view, *m* is the number of other individuals in the field of view, and $\:\stackrel{-}{\boldsymbol{v}}$ is the average velocity vector of other individuals in the field of view, calculated:

11 $$\:\stackrel{-}{\boldsymbol{v}}=\frac{\sum\:_{k=1}^{m}{\boldsymbol{v}}_{k}}{m}$$

where ***v***_*k*_ is the velocity vector of the *k*^th^ individual in the field of view, and *m* is the number of other individuals in the field of view.

In this simulation, encounter between the fish model and feed model was defined as feed intake. The number of feed pellets encountered by each individual, $\:{{N}_{f}}^{i},$ was counted during the behavioral simulation. In the simulation, the number of pellets supplied per day was calculated by dividing the daily feed total by the weight of a single pellet. The encountered feed was immediately deleted from the simulation. This prevented multiple consumption of the same pellet. As a result, the feed intake weight, $\:{S}_{n}^{i}$, was calculated after the behavioral simulation:

12 $$\:{S}_{n}^{i}={{N}_{f}}^{i}\bullet\:{W}_{f}$$

where $\:{S}_{n}^{i}$ is the feed intake weight of the *i*^th^ individual on the *n*^th^ day, and *W*_*f*_ is the weight of one pellet of feed.

In the simulation, fish behavior was divided into standard and feeding modes. If the tank contained feed and the feed intake weight $\:{S}_{n}^{i}$ was lower than the maximum feed intake weight *S*_*max*_, then the individual was determined to be in feeding mode. Here, *S*_*max*_ was 4% of the body mass^16^. Otherwise, the individual was in standard mode.

The strength of each force was determined by the weight coefficients, *w*_*n*_ (Table 1), which were fixed except for approaching feed (*w*_*feed*_), which was determined by the mode of the individual. In this study, we set *w*_*feed*_ to 3.0 for feeding mode and 0.0 for standard mode^16^.

The maximum swimming velocity magnitude $\:{\left|\boldsymbol{v}\right|}_{max}$ was defined to consider the limit of a fish’s swimming ability. If the magnitude of the velocity in Eq. (1), $\:\left|\boldsymbol{v}\right|$, exceeded the maximum velocity magnitude, then the swimming velocity vector was corrected:

13 $$\:\boldsymbol{v}={\left|\boldsymbol{v}\right|}_{max}\bullet\:{\boldsymbol{e}}_{v}$$

where **e**_*v*_ is the unit vector of the swimming velocity vector ***v***. The maximum swimming velocity, $\:{\left|\boldsymbol{v}\right|}_{max}$ was proportional to total length (TL):

14 $$\:{\left|\boldsymbol{v}\right|}_{max}={C}_{maxvel}\bullet\:{TL}_{n}^{i}$$

where *C*_*maxvel*_ is the coefficient of maximum velocity magnitude (Table 1), which was modified by the swimming mode: *C*_*maxvel*_ = 1.5 for standard mode^21^ and *C*_*maxvel*_ = 7.0 for feeding mode^22^.

**Table 1:** Weight coefficients for each force vector and coefficients of maximum velocity for each swimming mode^16^.

	Interpretation	Standard mode	Feeding mode
*w*_*separation*_	Weight coefficient for separation behavior	1.0	1.0
*w*_*cohesion*_	Weight coefficient for cohesion behavior	0.2	0.2
*w*_*alignment*_	Weight coefficient for alignment behavior	0.2	0.2
*w*_*bound*_	Weight coefficient for boundary avoidance	1.5	1.5
*w*_*inertia*_	Weight coefficient of inertia	0.2	0.2
*w*_*feed*_	Weight coefficient for approaching feed	0.0	3.0
*w*_*random*_	Weight coefficient for random movement	0.2	0.2
*C*_*maxvel*_	Coefficient for maximum swimming velocity	1.5	7.0

#### Growth simulation

The growth model used in this study was based on a DEB model^10^, which considers physiological processes such as metabolism, assimilation, and excretion to simulate the growth of a target organism. We ignored terms related to reproduction and maturation, and added variables for stomach content based on previous studies^11,15^ according to the following differential equations:

15 $$\:\frac{d{G}_{n}^{i}\:}{dt}=\dot{k}\left(T\right)\left(\dot{{p}_{x}}-\dot{{p}_{a}}\right)$$

16 $$\:\frac{d{E}_{n}^{i}}{dt}=\dot{k}\left(T\right)\left({\kappa\:}_{x}\dot{{p}_{a}}-\dot{{p}_{c}}\right)$$

17 $$\:\frac{d{V}_{n}^{i}}{dt}=\dot{k}\left(T\right)\frac{\kappa\:\dot{{p}_{c}}-\dot{{p}_{s}}}{\left[{E}_{G}\right]}$$

where $\:{G}_{n}^{i}$, $\:{E}_{n}^{i}$, and $\:{V}_{n}^{i}$ represent the gut contents (J), energy in reserve (J), and structural body volume of the *i*^th^ individual on the n^*th*^ day, respectively, and $\:\dot{k}\left(T\right)$ is the rate of a physiological process at temperature *T*, expressed based on the Arrhenius equation:

18 $$\:\dot{k}\left(T\right)={\dot{k}}_{1}exp\left(\frac{{T}_{A}}{{T}_{1}}-\frac{{T}_{A}}{T}\right)$$

where $\:{\dot{k}}_{1}$ is the rate at the reference temperature (T_1_ = 293.15 K), $\:\dot{p}$ is the mass flux of each type of organic matter; $\:\dot{{p}_{x}}$ is food, $\:\dot{{p}_{a}}$ is assimilation, $\:\dot{{p}_{c}}$ is mobilization, and $\:\dot{{p}_{s}}$ is somatic maintenance. These mass flux processes are calculated:

19 $$\:\dot{{p}_{x}}=\:{S}_{n}^{i}{E}_{f}$$

20 $$\:\dot{{p}_{a}}={k}_{g}{G}_{n}^{i}$$

21 $$\:\dot{{p}_{c}}=\frac{{E}_{n}^{i}/{V}_{n}^{i}\left(\left[{E}_{G}\right]\dot{v}{{V}_{n}^{i}}^{\frac{2}{3}}+\left[\dot{{p}_{M}}\right]{V}_{n}^{i}\right)}{\left[{E}_{G}\right]+\kappa\:{E}_{n}^{i}/{V}_{n}^{i}}$$

22 $$\:\dot{{p}_{s}}=\left[\dot{{p}_{M}}\right]{V}_{n}^{i}.$$

The fish mass $\:{W}_{n}^{i}$ was calculated:

23 $$\:{W}_{n}^{i}={d}_{Vw}+{E}_{n}^{i}\frac{{w}_{Ed}{d}_{Vw}}{{\mu\:}_{E}{d}_{Vd}}\:.$$

Finally, the fork length $\:{FL}_{n}^{i}$ was calculated based on the following allometric equation:

24 $$\:{W}_{n}^{i}=a{{FL}_{n}^{i}}^{b}\:\iff\:\:{FL}_{n}^{i}={\left(\frac{{W}_{n}^{i}}{a}\right)}^{\frac{1}{b}}$$

where *a* and *b* are allometric parameters determined in our rearing experiment. Other parameters used in Eqs. (15–24) are listed in Table 2.

**Table 2:** Growth model parameters based on an energy budget (DEB).

Symbol	Value	Unit	Interpretation	Ref.
Temperature parameters in Eq. (18)				
*T*_*A*_	8000	K	Arrhenius temperature	1
*T*_1_	293.15	K	Reference temperature	1
Core parameters for mass fluxes in Eqs. (19)–(22)				
*κ*_*x*_	0.8	-	Digestion efficiency of food to reserves	1
*κ*	0.61916	-	Allocation fraction to soma	1
[*E*_*G*_]	5267.56	J/cm^3^	Specific cost for structure	1
*k*_*g*_	20	-	Coefficient for assimilation	2
$\:\dot{v}$	0.032453	cm/d	Energy conductance	1
$\:\left[\dot{{p}_{M}}\right]$	343.884	J/cm_3_/d	Volume-specific somatic maintenance rate	1
Weight conversion parameters in Eq. (23).				
$\:{w}_{Ed}$	23.9	g/mol	Molecular weight of dry mass for reserve energy	1
$\:{d}_{Vw}$	1.0	g/cm^3^	Specific density of wet structure	3
$\:{\mu\:}_{E}$	550,000	J/mol	Chemical potentials for reserve energy	1
$\:{d}_{Vd}$	0.2	g/cm^3^	Specific density of dry structure	3

^1^ Add-my-Pet (http://www.bio.vu.nl/thb/deb/deblab/add_my_pet. Last accessed: August 15, 2025).

^2^ Alver et al.^12^.

^3^ Stavrakidis–Zachou et al.^24^.

#### Workflow of the simulation

**Fig. 3:**
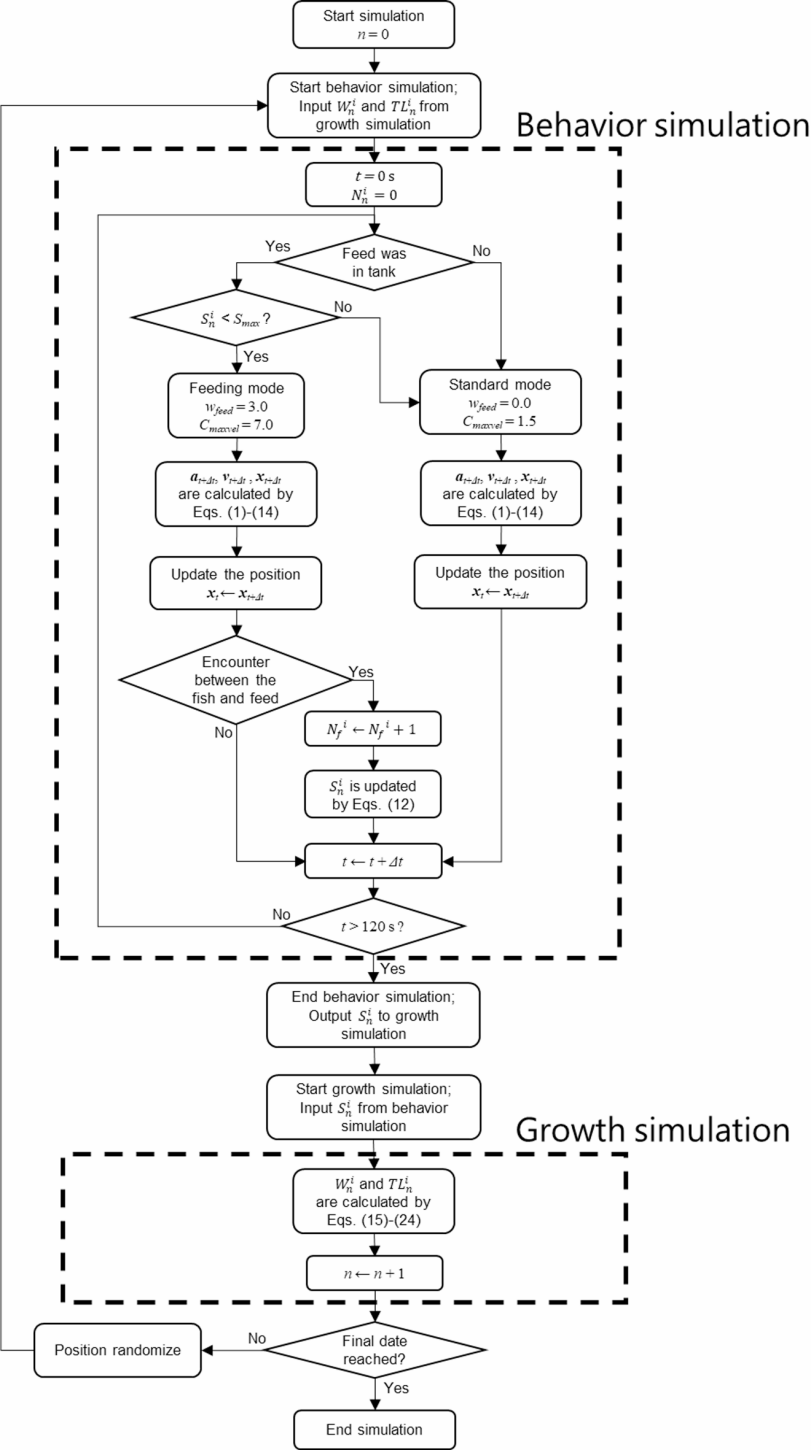
Detailed flow chart of the proposed simulation.

A detailed flow chart of the simulation for individual fish is shown in Fig. 3. The model included a behavioral simulation, in which actions are updated every 0.05 s based on swimming behavior governed by Eqs. (1–14). The behavioral simulation was conducted for 120 s, with feed distributed at 60 s. The time durations were set after we confirmed that all feeding behaviors of the simulated fish had been completed. After the behavioral simulation, the individual feed intake amount, $\:{S}_{n}^{i}$, was evaluated and passed as an argument to the growth simulation, where it was used to determine the body mass $\:{W}_{n}^{i}$ and fork length $\:{FL}_{n}^{i}$ of each individual by solving the system of differential equations in Eqs. (15–24) using the 4th -order Runge–Kutta method.

The body mass $\:{W}_{n}^{i}$, fork length $\:{FL}_{n}^{i}$, gut content $\:{G}_{n}^{i}$, structural volume $\:{V}_{n}^{i}$, and internal energy $\:{E}_{n}^{i}$ calculated in the growth simulation were passed on to the next step (*n* + 1).

In the simulation, one day corresponded to 120 s of behavioral simulation followed by growth simulation. To avoid unintended carry-over of spatial patterns, the swimming positions of all individuals were randomized at the end of the growth simulation, and the behavioral simulation for the next day then started. Similar randomization procedures were tested in a previous study^16^, and no substantial differences in growth outcomes were observed. This process is repeated iteratively until the end date, and the final body mass and fork length are simulated.

### Rearing experiment for simulation validation

To validate the proposed simulation model, we conducted a live rainbow trout rearing experiment in a circular tank with a nominal capacity of 500 L and an inner diameter of 0.9 m. The water depth during the experiment was set to 0.6 m. The experiment was conducted at the Nanae Freshwater Field Station and Science Center for the Northern Biosphere at Hokkaido University. The rainbow trout used in this study were hatched and reared at the same facility. The initial body weight was 2.94 ± 1.05 g (mean ± s.d.) and the initial fork length was 5.68 ± 0.69 cm (mean ± s.d.). The water temperature was maintained at 10 °C. As the experiment targeted pre-smolt individuals, it was conducted in freshwater. The experiment was conducted from 30 March to 19 October, 2022, spanning 203 days. During the experimental period, feeding rates (e.g., % body weight per day) were not predefined. Instead, excess feed was provided, and the amount of feed consumed was recorded daily. These experimentally recorded daily feeding amounts were used as input conditions for the simulation.

**Fig. 4:**
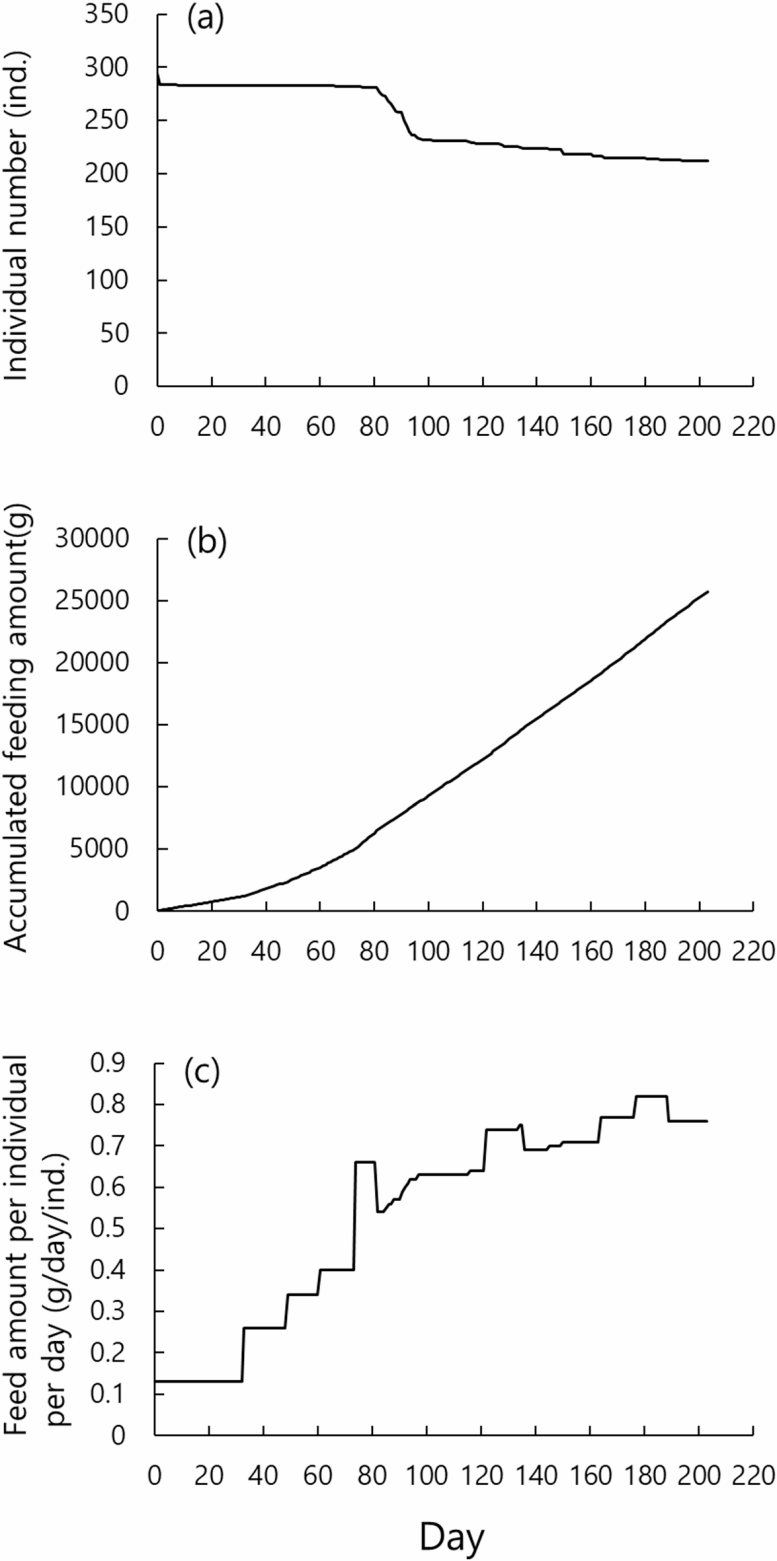
(**a**) Individual number, (**b**) accumulated feeding amount, and (**c**) daily feeding amount per individual during the rearing experiment.

Smoltification occurred in some individuals during the experiment. However, the freshwater conditions were maintained to validate the simulation. These smoltified individuals experienced osmotic stress, which likely contributed to the observed mortality. Such mortality was therefore physiologically driven rather than a result of disease or inadequate handling. Consequently, the number of individuals in the tank was initially 331, decreasing to 212 by the end of the experiment as individual fish died. The daily feeding rate per individual throughout the experiment, calculated from the total feeding amount and number of individuals, is shown in Fig. 4. We multiplied the daily feeding amount per individual by the total number of individuals remaining at the end of the experiment (*n* = 212), for use in the simulation model. The type of feed was changed according to the growth stages of the fish. The detailed properties of the feed provided to the fish are provided in Table 3.

**Table 3:** Description of feed used in the rearing experiment and simulation.

Rearing period	Feed	Calorie of feed (J/g)	Weight of one pellet W_f_ (g)
Day 1–32	Masu-Chigyo super EPC-1 (Marubeni Nissin Feed Co., Ltd.)	14961.28	0.002
Day 33–60	Masu-Chigyo super EPC-2 (Marubeni Nissin Feed Co., Ltd.)	14961.28	0.005
Day 61–73	Masu-Kokei 3 (Marubeni Nissin Feed Co., Ltd.)	14359.29	0.022
Day 74–176	Masu-Kokei 4 (Marubeni Nissin Feed Co., Ltd.)	14359.29	0.072
Day 177–203	Masu-Kokei 5 (Marubeni Nissin Feed Co., Ltd.)	14359.29	0.110

**Fig. 5:**
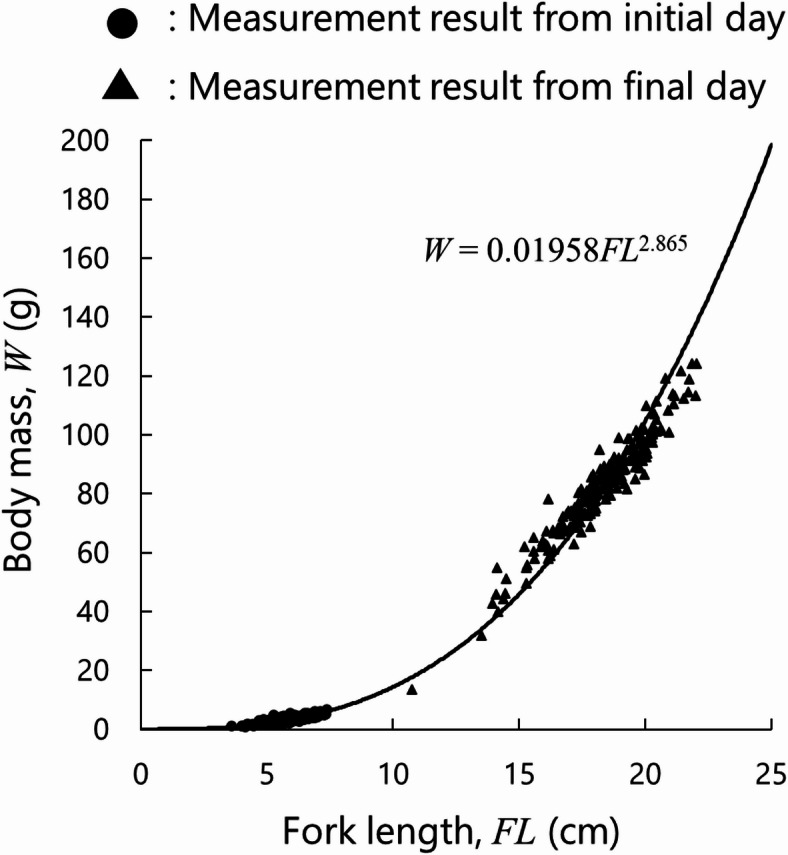
Relationship between fish body mass and fork length, from which allometry equation parameters were estimated (*a* = 0.01958; *b* = 2.865).

At the beginning and end of the experimental period, all fish were anesthetized using FA100 (Bussan Animal Health Co., Ltd, Osaka, Japan), an eugenol-based anesthetic, at a concentration of approximately 1:5,000 (v/v), following the manufacturer’s instructions. After being anesthetized, individual fish body mass and fork length were measured. During the experimental period, the fork length of some sampled fish was measured non-invasively approximately once per month using a stereo camera (UC-150) developed by Furuno Electric Co., Ltd^23^. Because the fish body mass could not be measured non-invasively during the experimental period, it was estimated using the allometric relationship given in Eq. (24) based on individual measurements conducted at the beginning and end of the experiment. The relationship between body mass (g) and fork length (cm) is shown in Fig. 5. The parameters of the equation were determined based on the figure using the least-squares method (*a* = 0.01958; *b* = 2.865).

The Animal Care and Use Committee of the Faculty of Fisheries Sciences, Hokkaido University, waived the need for formal approval of the live rearing fish experiments. Nevertheless, all methods adhered to the relevant institutional and national guidelines and regulations. All methods are reported in line with the ARRIVE guidelines.

### Simulation model setup

The conditions for the simulation were decided based on the rearing experiment. A simulated circular 500-L tank with a diameter of 0.9 m and depth of 0.6 m was used. The deaths of individual fish were not taken into account, such that the number of individuals remained constant, set at the total number of individuals on the final day of the rearing experiment (212 individuals). The feeding amount per individual (Fig. 4c) was multiplied by 212 to determine the feeding amount for the simulation. This value served as the input to the behavioral simulation. Feed pellets were released into the simulated tank and redistributed among individuals via pellet–fish encounter. The resulting individual feed intake was used for growth calculations. The types of feed used was set to match those used in the experiment (Table 3). The number of feed pellets provided was determined by dividing the set feeding amount by the weight of one pellet.

## Results

### Simulation overview

**Fig. 6:**
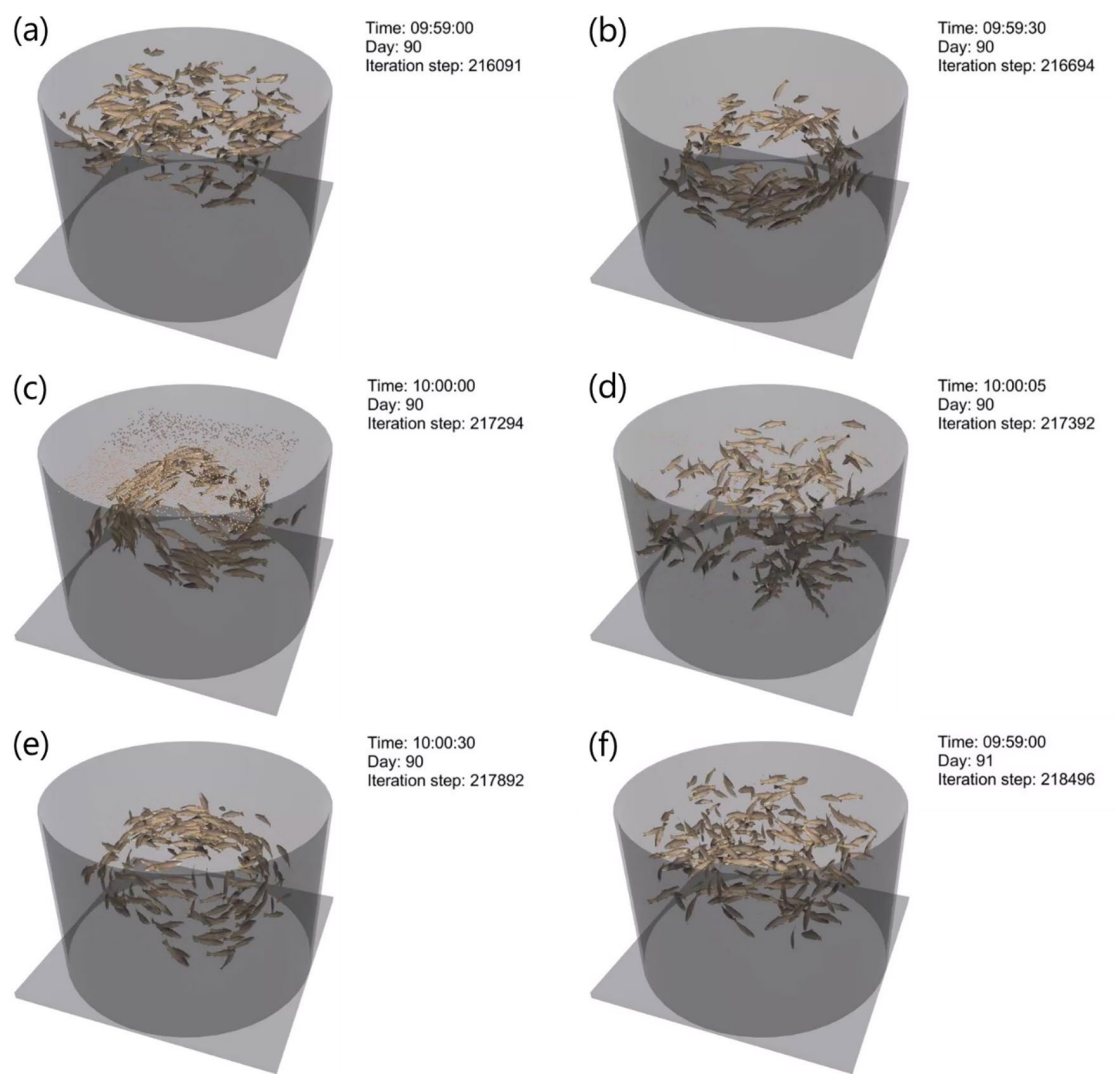
Overview of the simulation, showing day 90–91 as an example. (**a**, **b**) Simulated fish swim and form schools. (**c**) Feed is distributed at a set time (10:00). (**d**) Fish consume the feed. (**e**) After feeding, the fish swim freely. (**f**) At 120 s after the start of the behavior simulation, the simulation moves to the next day and the swimming positions of all individuals are randomized.

The simulation results are shown in Fig. 6 and an animation of the simulations is presented in the Supplementary Information. Figure 6 provides an overview of days 10–11 as an example. The simulated fish were randomly distributed in the tank at the start of day 10 (Fig. 6a). As time progressed, the fish simulated exhibited natural swimming patterns and flocking behaviors (Fig. 6b). At the set time, feed was released at the top of the tank (Fig. 6c), and the fish moved toward the feed (Fig. 6d). After feeding, the simulated fish resumed natural swimming patterns (Fig. 6e). After a growth update based on the feeding amount, the model then moved to the next day, again randomizing the fish swimming positions. This avoided unintended carry-over of spatial configurations (Fig. 6f).

### Comparison of simulation and experimental results

**Fig. 7:**
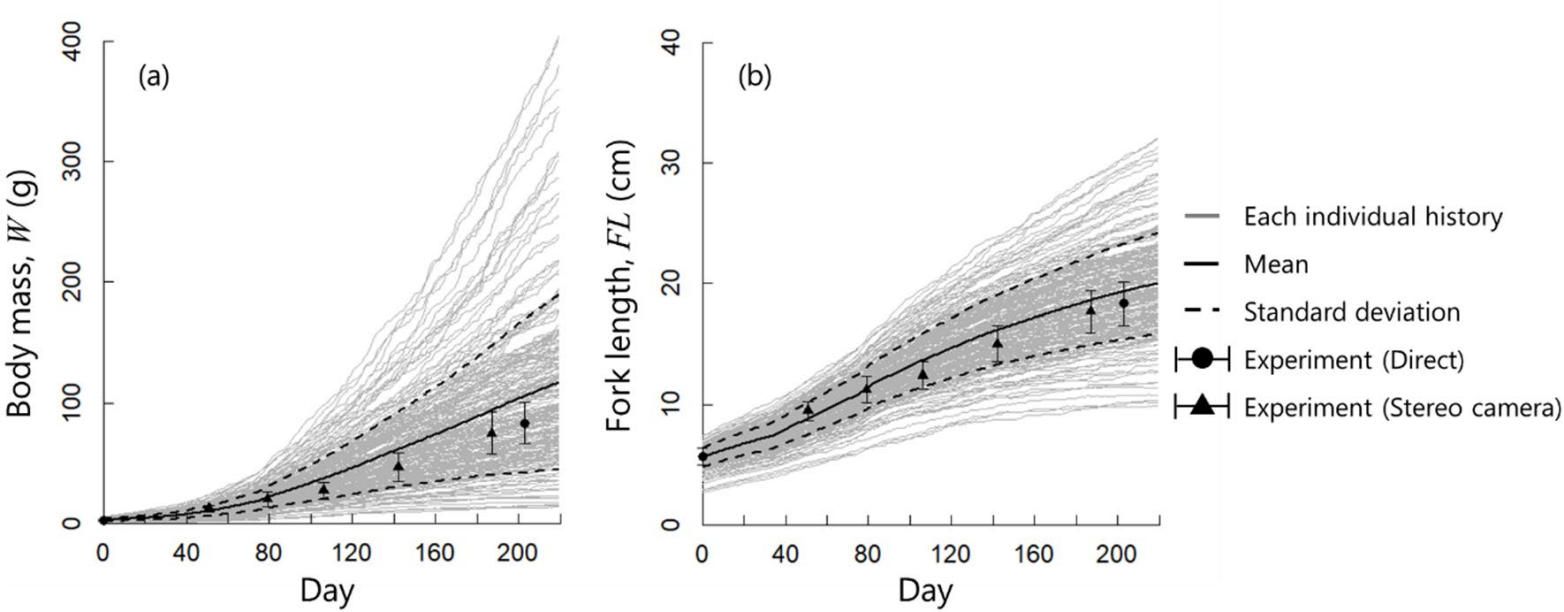
Simulation results for (**a**) body mass and (**b**) fork length, showing mean values from the rearing experiment for comparison. Circles indicate direct measurements, and triangles indicate non-invasive measurements obtained using a stereo camera. Gray, black, and dashed lines represent the growth trajectories of individual simulated fish, mean values, and standard deviation, respectively.

**Fig. 8:**
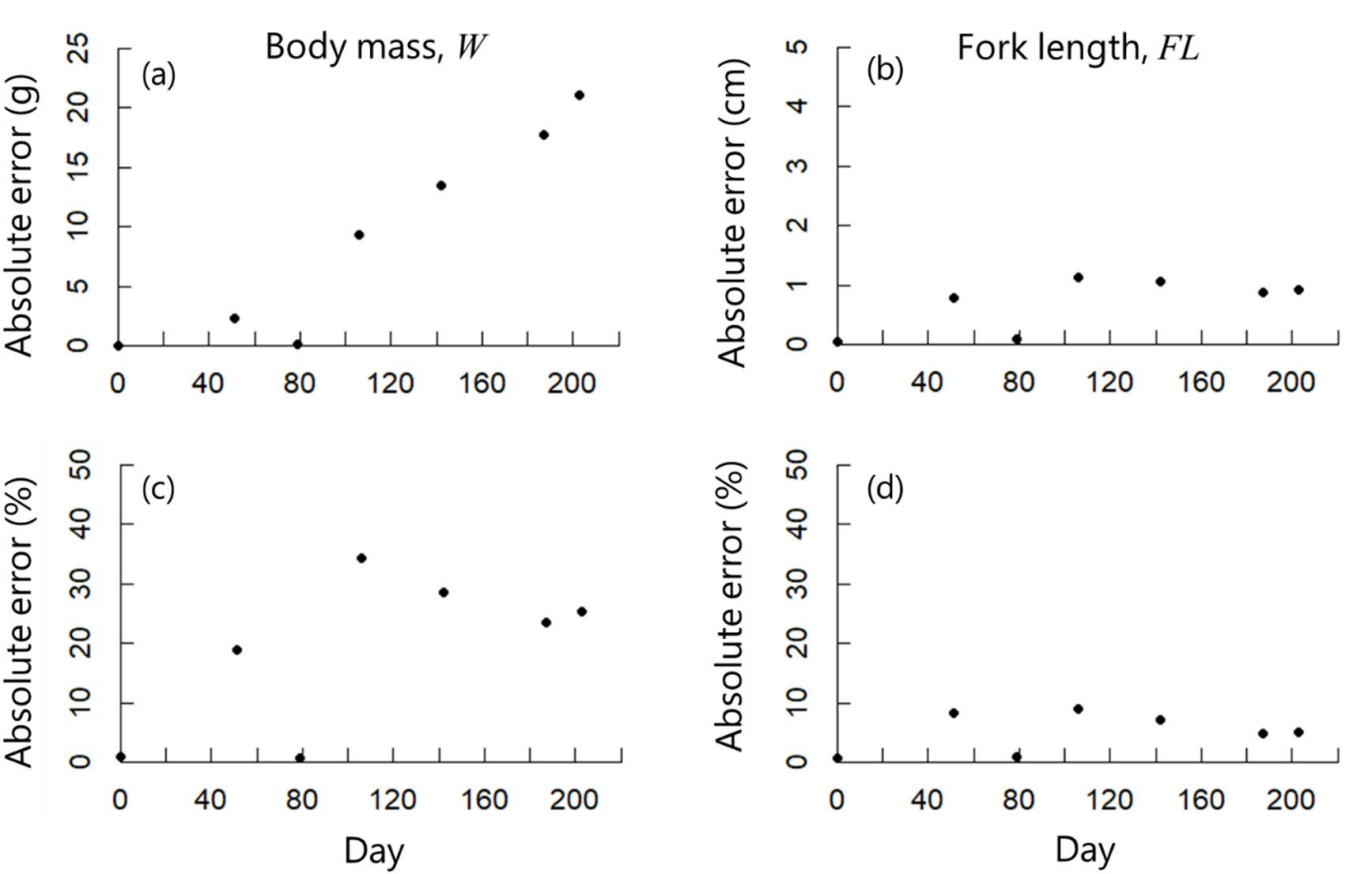
Differences between simulation and experimental results for (**a**) body mass (g) and (**b**) fork length (cm). Error rates (%) for (**c**) body mass and (**d**) fork length.

Estimated body mass and fork length values obtained from the simulation were compared with the experimental values (Fig. 7). The absolute error was calculated as |Simulation value – Experimental value|, and the absolute percentage error was calculated as |Simulation value – Experimental value| × 100/Experimental value (Fig. 8).

Estimated body mass increased exponentially throughout the simulation (Fig. 7a), and variability (standard deviation) also increased. The variability among individuals gradually increased over time in both the experiment and the simulation; however, the magnitude of the variability was larger in the simulation. Notably, the simulation values well reflected the experiment values in the early stage of rearing; however, the absolute error gradually increased after approximately 80 days (Fig. 8a). Overall, the simulation results were overestimated compared with the experimental values, with absolute error and absolute percentage error values of 19.0 g and 22.7%, respectively (Fig. 8a, c).

The simulated fork lengths increased linearly until day 120 and declined thereafter (Fig. 7b), with variation increasing continuously although to a lesser degree than the body mass. Similar trends were observed in the experimental fork length measurements, with a proportional increase early in the simulation followed by a decline. The absolute error between the simulation and experimental fork length values remained nearly constant throughout the simulation period (Fig. 8b). The absolute percentage error was lower for fork length than for body mass, within the range of 4–10% (Fig. 8d).

### Feeding amount sensitivity analysis

During the early rearing phase (days 0–79), the experimental FCR was 1.19, whereas the simulation yielded an FCR of 1.18, indicating good agreement between the two. However, over the entire rearing period (days 0–203), the experimental FCR was 1.34, whereas the simulated FCR was 1.06. This discrepancy is likely attributable to overestimation of mean body weight during the later stages of rearing, as shown in Fig. 8.

**Fig. 9:**
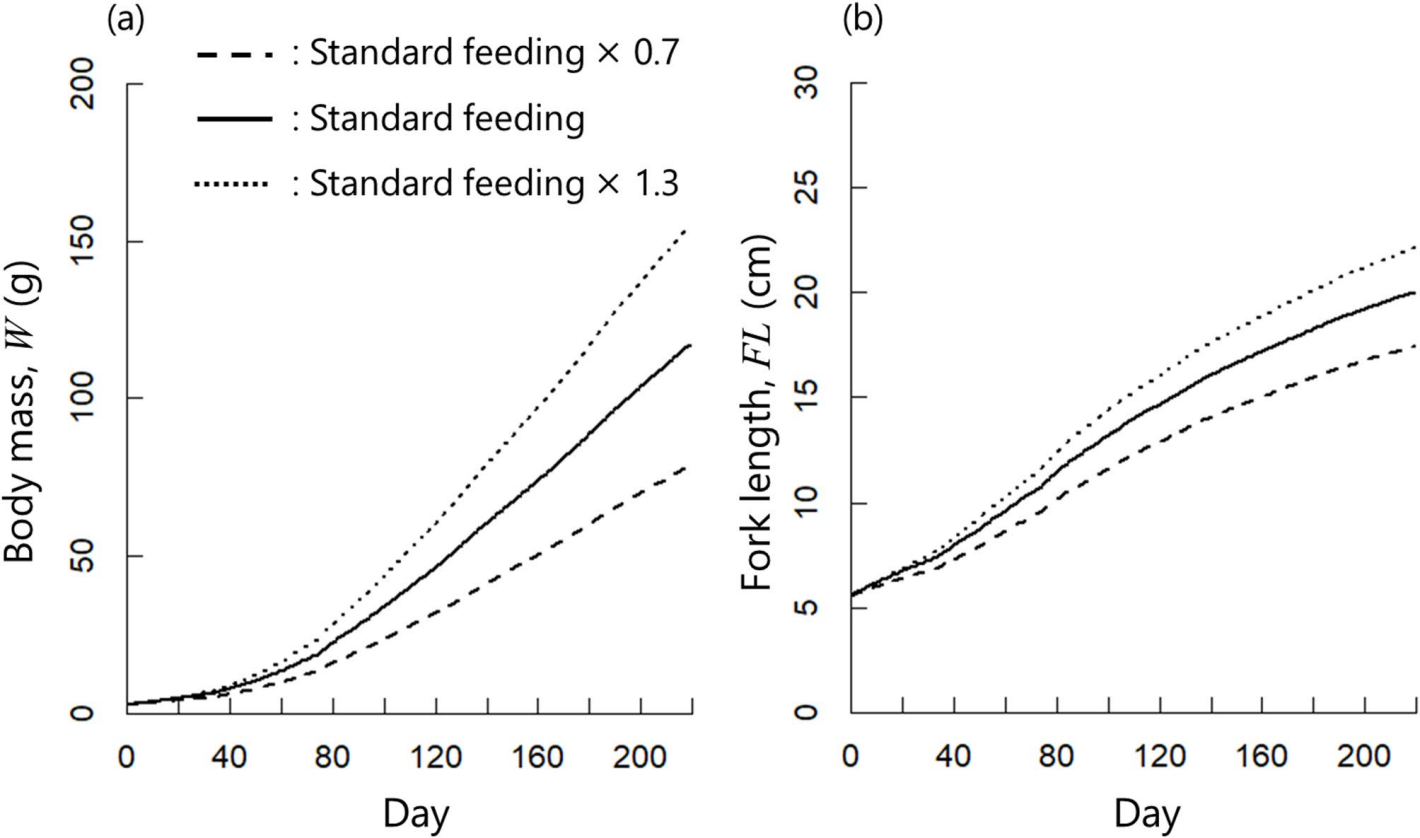
Simulation results for various feeding levels relative to the default settings of the simulation model. (**a**) Body mass. (**b**) Fork length.

**Fig. 10:**
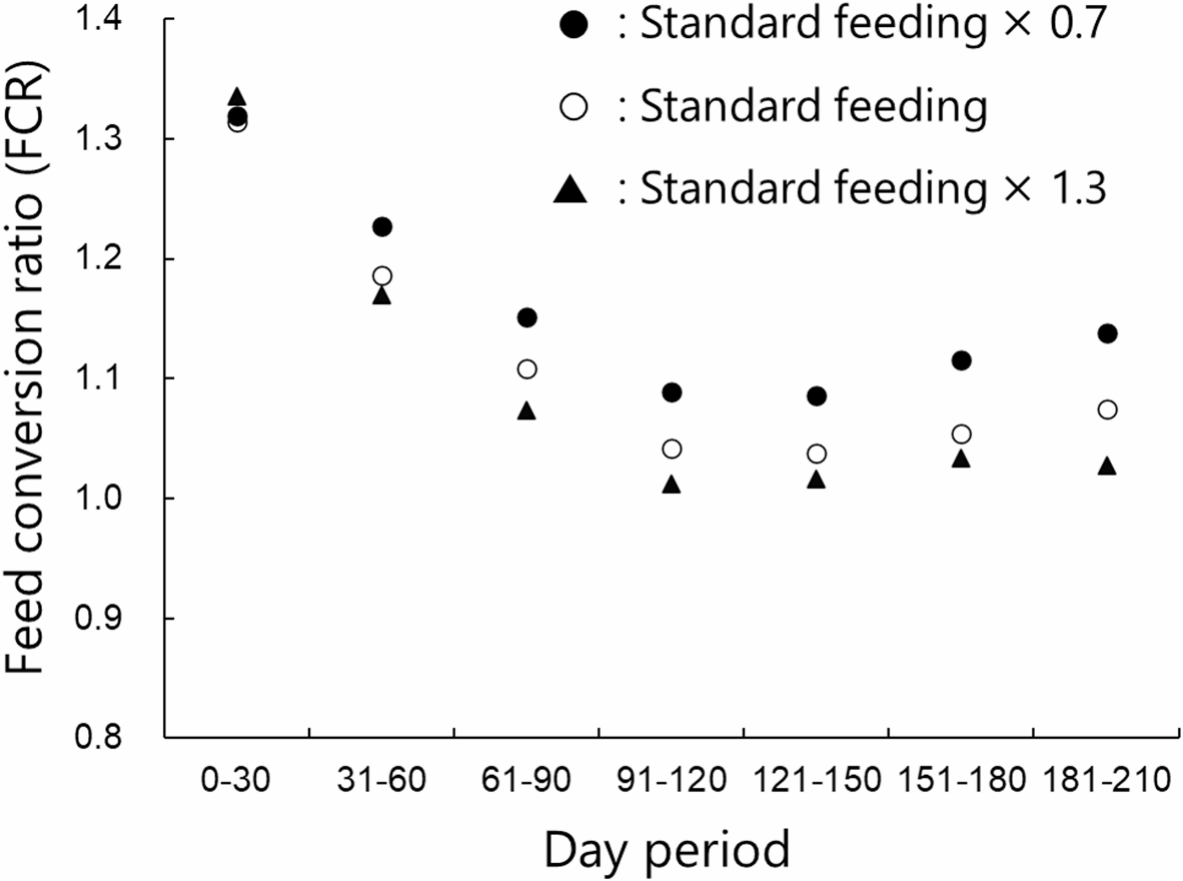
Feed conversion ratios (FCRs) for various feeding levels and growth stages.

To evaluate changes in growth based on the feeding amount, we performed a sensitivity analysis, setting the feeding amount at 0.7 and 1.3 times the standard feeding amount shown in Fig. 4(c). Here, the standard feeding amount was the experimentally measured daily feeding amount per individual, which served as the baseline input for the simulation. The mean values of body mass and fork length for each feeding amount are presented in Fig. 9. Changes in the feed conversion ratio (FCR, defined as the feeding amount/extent of growth) at 30-day intervals are shown in Fig. 10.

As shown in Fig. 9, the growth speed increased as the feeding amount increased. At day 200, the mean body masses were 70.2, 103.9 and 137.2 g for 0.7, 1.0 and 1.3 times the standard feeding amount, respectively. The FCR decreased as the feeding amount increased in the early simulation period (days 0–30), by 1.31, 1.25 and 1.31 for 0.7, 1.0 and 1.3 times the standard feeding amount, respectively. After the first 30 days, the opposite trend was observed. During the final period (days 181–210), the FCR was 1.13, 1.06 and 1.02 for 0.7, 1.0 and 1.3 times the default feeding amount, respectively.

## Discussion

In this study, we developed a simulation model that reproduced the fish rearing process and determined its suitability for aquaculture applications. Simulated and experimental values were compared to validate the proposed model. The model reproduced trends in both the body mass and fork length of fish reared under experimental conditions. Although the mortality rate was relatively high, this was primarily attributable to smoltification under freshwater conditions. As the experiment was designed to validate the simulation framework rather than to investigate biological mortality processes, this limitation does not invalidate the comparison. In addition, we performed supplementary simulations assuming no mortality, using the initial number of individuals (331 fish). These results are presented in Supplemental Information. No significant differences were observed in either the mean body mass or the fork length between the two simulations with and without mortality (Wilcoxon rank-sum test, *p* = 0.335 for body mass and *p* = 0.335 for fork length). We conclude, therefore, that the observed mortality did not affect the validation results of this study.

Notably, the accuracy of the body mass estimation was high up to day 80; however, the error increased with simulation time, reaching 22.7% on the final day (day 203). This growth overestimation propagated to the feed-related metrics, associated with discrepancies in the simulated FCR, compared to those of the experimental measurements.

One possible reason for the increasing error rate is the non-invasive method used to measure fish body mass in the rearing experiment, which relied on a stereo camera. The stereo camera measured fork length accurately but could not measure body mass directly. Therefore, we estimated body mass from the fork length measurements using the allometric relationship shown in Eq. (24). As indicated in Fig. 5, the discrepancy between the estimated and measured values increased gradually during the experiment, suggesting that some estimation error was unavoidable. Although this measurement error is unlikely to be the primary cause of the overall deviation, it may have contributed to a portion of the variability observed in the comparison between the experiment and the simulation. Another possible reason for the increasing error with simulation time is that density effects were not considered in the proposed simulation. In this experiment, the initial stocking density was 0.19%, and attained 3.5% at the end of the rearing period (day 203). Generally, fish growth rates decrease as the rearing density increases. Therefore, the lower growth rate observed in the later stages of the rearing period is likely attributable to the increased stocking density. This phenomenon may have contributed to the overestimated simulation results. In this study, density-dependent reductions in growth are primarily viewed as a consequence of water-quality degradation, particularly decreases in dissolved oxygen (DO). Although the model considers temperature, DO dynamics and their effects on feeding and metabolism are not included. This omission likely contributed to the discrepancy between the experimental and simulated growth during the later rearing stages. Future work will incorporate DO-dependent physiological processes into the model.

Furthermore, we applied the default parameters of the DEB, which may require adjustment. Fish growth can vary depending on the seedling lot or other biological factors. To overcome such variation, Stavrakidis–Zachou et al.^24^ proposed a method to adjust DEB model parameters for the European sea bass *Dicentrarchus labrax*. In a future study, we will aim to simulate body mass more accurately by taking all of these factors into account.

The variability among individuals gradually increased over time in both the experiment and the simulation; however, the magnitude of this variability was larger in the simulation. In the current model, a feeding event is defined as a direct encounter between a fish model and a feed pellet model. As shown in Eq. (14), larger individuals are also assigned higher swimming speeds. Consequently, faster-swimming fish tend to reach the feeding area earlier and encounter more pellets, which may result in those individuals being treated as having consumed more feed than would realistically be possible. Given this simplified representation of pellet–fish encounters, large individuals may effectively monopolize the feed, while smaller individuals may have fewer feeding opportunities, leading to a greater divergence in individual growth in the simulation than in the experiment.

In addition, although an upper limit for daily feed intake was imposed in the model, this constraint was introduced to account for potential overconsumption at the individual level in an agent-based framework. In the rearing experiment, the average feeding amount was already sufficient at the population level; however, feeding opportunities in the simulation may still allow certain individuals to consume more feed than would be realistic, particularly under increased feed delivery conditions. This limitation may have contributed to overestimation of growth at higher feeding scenarios and to the enhanced individual variability observed in the simulation.

To address this issue, future work should incorporate a more detailed feeding behavioral model based on observational data, such as explicit modeling of feeding rates, ingestion probability, and the distinction between pellet encounter and actual ingestion.

The major advantage of the proposed method is that it can simulate the growth trajectories of individual fish. Our results revealed that a few individuals were exceptionally large or small; fish that were larger in the initial stages continued to grow to exceptional sizes, while the smaller fish remained smaller throughout the simulation (Fig. 7). A similar trend was reported in a previous study^16^, which highlighted that a narrow feeding area would further accentuate this trend. Under these circumstances, initial size may have a significant impact on the growth rate and final size of an individual. Therefore, using simulations can allow for more accurate predictions of variability in growth rates among individuals in aquaculture farms. Additionally, understanding the growth trajectory of every individual would be beneficial for feed management and determining the optimal harvest time. Individuals that are too large or too small can complicate processing and marketing operations in aquaculture farm management as they reduce size uniformity and render it difficult to predict market prices. Therefore, such individuals are typically removed from the population. Thus, ascertaining the growth status of such individuals in real time allows for informed decisions regarding feed management and harvest timing. However, as discussed above, the present model reproduces these individual differences at only a qualitative level, and the magnitude of variability is overestimated. We expect that incorporation of a more realistic feeding process—particularly improvements in how feeding events are defined—will enhance the ability of the model to match the observed size distribution quantitatively and thereby increase its practical usefulness for aquaculture management.

Our sensitivity analysis of the simulated feeding amounts indicated that the fish had a larger final size when the feeding amount was large. However, the opposite trend was observed for the FCR. The FCR is defined as the feed weight required to increase the body mass by 1 kg; therefore, a low FCR indicates a higher feeding efficiency. During the early stage of the simulation (days 0–30), the default feeding amount was the most effective, with an FCR of 1.25. However, during the subsequent period (days 30–), the 1.3× feeding scenario resulted in the lowest FCR. The simulation results demonstrate that FCR varies according to the fish growth stage. According to Hasan^3^, approximately 60% of aquaculture costs are attributed to feed. Thus, optimizing feed efficiency is a crucial issue for aquaculture farms. The proposed simulation considers feeding efficiency and can forecast future aquaculture conditions, making it a valuable tool for decision-making in aquaculture farms.

The proposed simulation model predicted individual fish growth trajectories and assessed the impacts of varying feeding levels in aquaculture systems. The growth of rainbow trout was simulated by the proposed method under controlled conditions, with reasonably accurate results obtained during the early stages of the simulation. However, an increasing difference between simulation and experimental results was observed as the simulation time was extended. To address this problem, the simulation model should take density effects into account, and the parameters should be adjusted; these changes are anticipated to improve the accuracy of the proposed model. The proposed simulation represents a useful tool for aquaculture decision-making, allowing managers to explore the effects of different feeding strategies, optimize growth, and enhance feed efficiency. Furthermore, the model may be applied to other aquaculture species besides rainbow trout. This study contributes a simulation-based approach for better understanding and management of aquaculture systems.

## Supplementary Information

Below is the link to the electronic supplementary material.

Supplementary Material 1

Supplementary Material 2

## Data Availability

The datasets generated during and/or analysed during the current study are available from the corresponding author on reasonable request.
