# Supplementary material for: An aquaculture simulator for rainbow trout (Oncorhynchus mykiss) based on a fish schooling behavioral model and a dynamic energy budget
Source: Sci Rep. 2026 Feb 7;16:7706. doi: 10.1038/s41598-026-39028-y (PMC12946377; doi:10.1038/s41598-026-39028-y)
Supplement: Supplementary file 2 — Supplementary Material 2 [file 41598_2026_39028_MOESM2_ESM.docx]

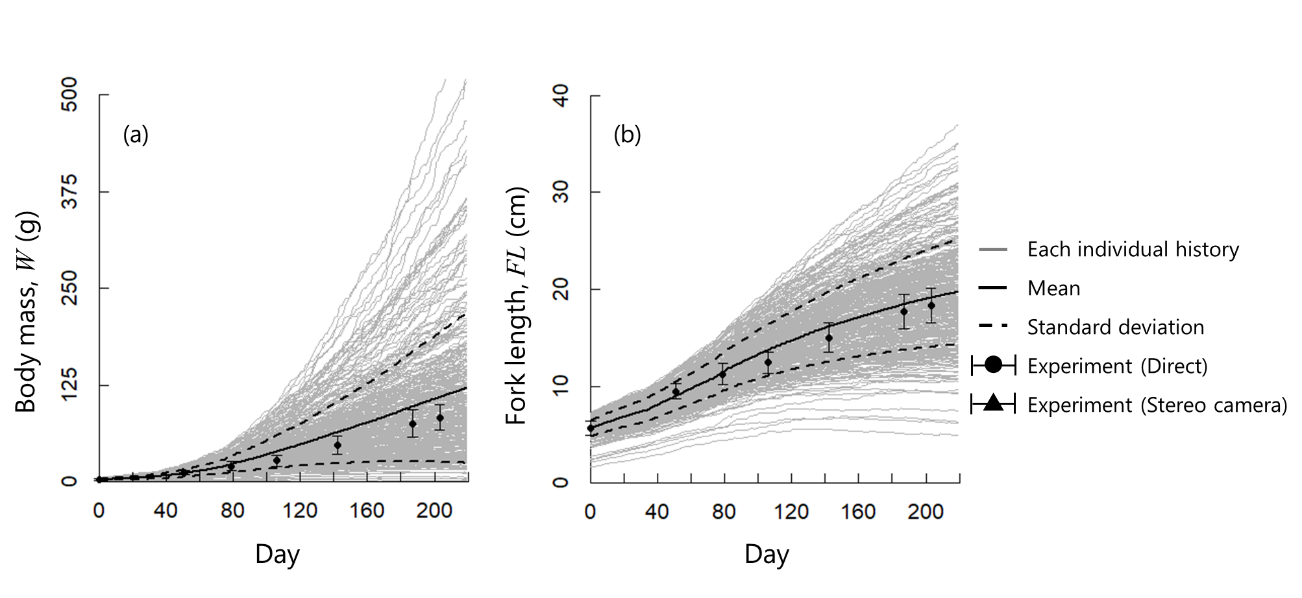


Supplementary Fig. S1. Simulation results assuming no mortality (331 individuals) for (a) body mass and (b) fork length are shown, together with mean values from the rearing experiment for comparison. Circles indicate direct measurements, and triangles indicate non-invasive measurements obtained using a stereo camera. Gray lines represent growth trajectories of individual simulated fish, while black solid and dashed lines indicate the mean values and standard deviation, respectively.

The simulation assuming no mortality resulted in slightly higher mean values, and the standard deviation was also larger. However, no statistically significant difference was detected between the no-mortality simulation (331 individuals) and the simulation shown in Fig. 8 (212 individuals) (Wilcoxon rank-sum test, *p* = 0.335 for body mass and *p* = 0.335 for fork length).
